# Persisting inhibition biases efficient rule inference under uncertainty

**DOI:** 10.3389/fpsyg.2024.1308636

**Published:** 2024-02-23

**Authors:** Pierpaolo Zivi, Anna Zigrino, Alessandro Couyoumdjian, Fabio Ferlazzo, Stefano Sdoia

**Affiliations:** Department of Psychology, Sapienza University of Rome, Rome, Italy

**Keywords:** inhibition, task set, set shifting, task switching, rule inference, cognitive control, decision making

## Abstract

**Introduction:**

Task set inhibition supports optimal switching among tasks by actively suppressing the interference from recently executed competing task sets. It is typically studied in cued task-switching paradigms where there is no uncertainty about the task set or rule to prepare for on each trial. While inhibition has been shown to influence the speed and the accuracy of task execution, affecting task set retrieval, preparation, or implementation in conditions of task set switching, it remains uninvestigated whether it also affects rule selection under uncertainty.

**Methods:**

We implemented an ad-hoc four-rule card sorting task and categorized the rules selected by participants after a rule shift according to the recency of their last usage. We included a measure of working memory capacity (WMC) to control for its involvement in the rule selection process.

**Results:**

Participants exhibited a reduced preference for recently abandoned rules than less recently abandoned ones. Furthermore, we found that such a preference was not associated with WMC.

**Discussion:**

The results suggest that decision-making processes underlying rule inference and selection may be influenced by task-set inhibition, configuring as a conflict adjustment mechanism to the sequential history of rules application.

## 1 Introduction

In many everyday circumstances, individuals must usually choose and pursue one among different viable action courses under a certain degree of uncertainty. Whatever the choice, individuals will have to choose the appropriate task set that will guide action selection. A task set is the ensemble of cognitive processes and representations defining a procedural schema (Monsell, 2003). A task set consists of goal-relevant parameters of the ongoing performance (Monsell, 2017), such as stimulus–response mappings (e.g., “if the semaphore light turns red, then stop”) or task-relevant stimulus attributes (e.g., “pay attention to the color of the semaphore lights, no matter their shape”). Typically, experiments in cognitive psychology comprise extensive instructions and overtrained tasks, allowing for the establishment of the task set in terms of rules to follow to achieve task goals. Individuals are required to rapidly and appropriately change or maintain the task rules depending on whether environmental demands change, proving the flexible and controlled regulation of task sets (Meiran, 2000; Monsell, 2017).

Literature has extensively shown that several mechanisms acting at different levels and stages of task processing (Koch et al., 2010) support task-set switching by counteracting proactive interference (Allport and Wylie, 1999) from old task sets. For instance, one of such mechanisms is the inhibition of competing (e.g., previously used) task sets, which has been hypothesized to be triggered during task-set switching (Mayr and Keele, 2000). Consequently, performing a task that has been recently abandoned (ABA task sequences), compared to performing a less recently abandoned one (CBA task sequences), typically results in slower and more error-prone performance (Mayr and Keele, 2000). Thus, a disadvantage rather than an advantage in repeating recently abandoned tasks (i.e., N-2) is usually observed. The difference in RTs (reaction times) and ERs (error rates) between the last trial of ABA and CBA sequences (the N–2 repetition cost) has been taken as a reliable indicator of the occurrence of task-set inhibition. Task-set inhibition represents a protective mechanism that is activated as soon as interference between task sets is detected (for a review, see Koch et al., 2010).

While it has been shown that inhibition can be triggered by conflicts arising at different levels of task set processing (Moretti et al., 2023a,b), e.g., at the level of stimulus features (Sdoia and Ferlazzo, 2008; Sdoia et al., 2022) or response-selection/execution (Schuch and Koch, 2003; Gade and Koch, 2007), less is known about the extent to which it can affect other, more voluntary components of behavior. For instance, it appears theoretically relevant to ascertain whether inhibition exclusively aids in executing tasks and applying rules or if it also plays a role in actively choosing to perform those tasks or use those rules. Recent evidence (Schuch and Dignath, 2021) has suggested that task-set inhibition may also guide decision-making. In their study, the authors (Schuch and Dignath, 2021) administered a cued task-switching paradigm with three different tasks and multivalent stimuli. After triplets of forced-choice trials (ABA or CBA sequences), a free-choice trial was presented. In such a trial, participants were required to choose which of the three tasks to perform as if they were mentally throwing dice (Arrington and Logan, 2004). The results showed that participants were biased in their decision strategy. Following an ABA forced-choice series, they showed a greater preference for selecting task C than tasks A and B, whereas following CBA sequences, they appeared to select tasks at random. Furthermore, participants were less likely to repeat the task (i.e., choosing the A task) after ABA than after CBA sequences. The authors argued that these results speak in favor of a conflict-adaptation hypothesis (Schuch et al., 2019), suggesting that the aftereffects of a proactive interference experienced in high conflict trials (ABA) affect subsequent performance (Schuch and Grange, 2015; Schuch et al., 2019). Within this view, task-set inhibition would be configured as an adjustment to the conflict arising when switching between two tasks (Sexton and Cooper, 2017; Schuch et al., 2019). The suggestion that decision-making processes can also be invested by a conflict-related adjustment is of great interest and merits further investigation (Schuch and Dignath, 2021).

Task-set inhibition processes are usually investigated through cued task-switching paradigms. In these paradigms, participants can fully anticipate the task set rule to prepare (e.g., press right for larger than 5 digits) and the effects of task set inhibition, inferred by the slowing of RTs and the proportion of ER, may represent a footprint of inefficient task-set retrieval, preparation, or implementation. Notably, decision-making in this situation is deterministic. Indeed, the chosen task is the one that will be performed: there is no uncertainty about the rules to follow. Nevertheless, information possessed by individuals about the rules governing everyday activities (e.g., how to perform a task) is typically limited, and feedback-driven trial-and-error strategies must be usually adopted to infer implicit task rules. Instead, in non-fully predictable environments individuals must deal with a certain level of uncertainty about which of several rules to follow in order to select the most appropriate set of actions. Under uncertainty, rule selection provides a frame for action selection. In this regard, it is currently not known how inhibitory processes acting at the task-set level may also support set-shifting when the relevant rules are implicit. However, to evaluate rule inference and selection processes, paradigms such as the Wisconsin Card Sorting Test (WCST; Grant and Berg, 1948) are traditionally used. On each trial of the WCST, participants are required to match one reference card with one among several (four in the original task) choice cards, according to different viable sorting rules (e.g., by the shape or the color of the symbols depicted on the cards). The correct sorting rule, however, changes unexpectedly after a run of trials and participants must use feedback received to choose if it is time to stick with their current sorting rule or to shift away from it. Importantly, when a shift in the sorting rule is required, participants are uncertain about which rule became relevant and must adopt a trial-and-error strategy. The cognitive processes underlying rule inference and selection in these tasks have received little attention, although they can provide crucial clues about how people deal with uncertainty independently from failures or errors in task set-related processes.

Errors in the WCST have been the subject of studies utilizing sorting tasks for both theoretical and clinical purposes (Barceló and Knight, 2002; Lange et al., 2016). In this regard, errors in set-shifting performance have been traditionally distinguished into errors stemming from a perseverative origin, which has been more closely linked to frontal areas (Milner, 1963) and errors with a non-perseverative nature. Perseverative errors (PEs) occur when individuals do not shift the sorting rule when they are required to do so (i.e., after negative feedback) and can be conceived as failures in the endogenous reconfiguration of a task set (Rogers and Monsell, 1995). Instead, non-perseverative errors (NPEs) may comprise both inefficient and efficient errors (Barceló, 1999; Barceló and Knight, 2002). On one hand, inefficient NPEs may occur if participants shift the sorting rule when not required to do so (i.e., after positive feedback), representing unsuccessful task-set maintenance (i.e., set-loss errors). Otherwise, inefficient NPEs may concern inefficient rule inference, e.g., when participants attempt to use a rule that has been already proven to be incorrect (Lange et al., 2016). On the other hand, efficient NPEs (i.e., when the rule is correctly shifted but the one attempted is not the correct one) can be considered normal outcomes due to the necessary trial-and-error strategy applied in response to the negative feedback acting as a switch signal. Therefore, efficient errors may represent an important glimpse into decision-making processes under uncertainty since they may provide useful information for rules’ preferences and biases. Since we were focused on evaluating rule inference strategies, efficient errors and correct sorts in the trials of maximum uncertainty (i.e., the trial immediately following the shift) have been considered together in the present work, to investigate whether the sequential history of previously relevant rules (e.g., ABA) may bias rule inference.

Previous research has shown that inhibitory and working memory processes are linked to rule maintenance and inference during rule shifting (Hartman et al., 2001; Buchsbaum et al., 2005; Steinmetz and Houssemand, 2011; Lange et al., 2016). These results have been explained (Lange et al., 2016) within the framework of the cognitive branching model (Koechlin et al., 1999; Koechlin and Hyafil, 2007; Charron and Koechlin, 2010), suggesting that, during task execution, the other viable action selection rules are processed and maintained in a pending state, ready to be used later. However, the increased information to be held by WM or reduced WM capacity is suggested to affect performance or rule selection due to the capacity limits of the inferential buffer suggested in the branching hypothesis (Lange et al., 2016). Indeed, it would be possible that individuals with low WMC are less able to keep rules active in WM, especially those used long before. Therefore, considering interindividual differences in WM capacity seems important in the investigation of the inhibitory processes involved in rule inference and selection.

The present study aimed to investigate whether task inhibition known to affect task set retrieval, preparation, or implementation in the condition of predictable task set switching also affects rule selection under uncertainty. To do so, we used an *ad-hoc* modified four-rules WCST paradigm. Since task-set inhibition is known to dissipate over time (Meiran, 2000), we categorized the participants’ choice on the first trial following the negative feedback acting as a switch signal according to the temporal distance between the current relevant rule and the last run of trials in which the chosen rule has been relevant. Indeed, in this trial, participants have been appropriately informed that the rule has changed and are maximally uncertain about which will be the next relevant rule. We hypothesized that participants’ trial and error strategies are affected by the recency of the last usage of the rules and expected three alternative results. In line with a task set rule persisting inhibition account, we should observe a preference for sorting rules that have not been recently relevant. Conversely, in line with a task set rule persisting activation account, the preference should be biased toward the most recent rules. Finally, we should observe no effects of recency if participants’ choice behavior is random. Since WM abilities can be implicated in at least the first two of our hypotheses and have been shown to account for performance in the WCST (Hartman et al., 2001), we included a direct measure of working memory capacity (WMC) to control for its possible role in the behavior of interest.

## 2 Materials and methods

### 2.1 Participants

Twenty-nine participants were recruited (Age: *M* = 24.17, SD 1.81; 20 females and 9 males). They were unaware of the study goals and hypotheses. The Institutional Review Board of the Department of Psychology of the Sapienza University of Rome approved the study, and all participants provided informed consent. The sample size was estimated using MorePower 6.0.4 (Campbell and Thompson, 2012). A sample of *N* = 22 was found to be enough to observe an effect size of 0.2 η^2^_p_ with a power of 0.9 and an α of 0.05 for the main effect in a one-way repeated measures ANOVA with recency as a 4-levels within-subjects factor.

### 2.2 Card sorting task

We administered via a laptop an *ad-hoc* modified computerized version of the Wisconsin Card Sorting Test (Grant and Berg, 1948) to assess rule-shifting processes. Stimuli were white cards depicting different items, which could vary according to four feature dimensions each comprising five levels: shape (squares, circles, triangles, stars, and crosses), color (red, green, yellow, pink, and blue), numerosity (from 1 to 5), and symbol position (center, up-right, up-left, down-right, and down-left).

**Figure 1:**
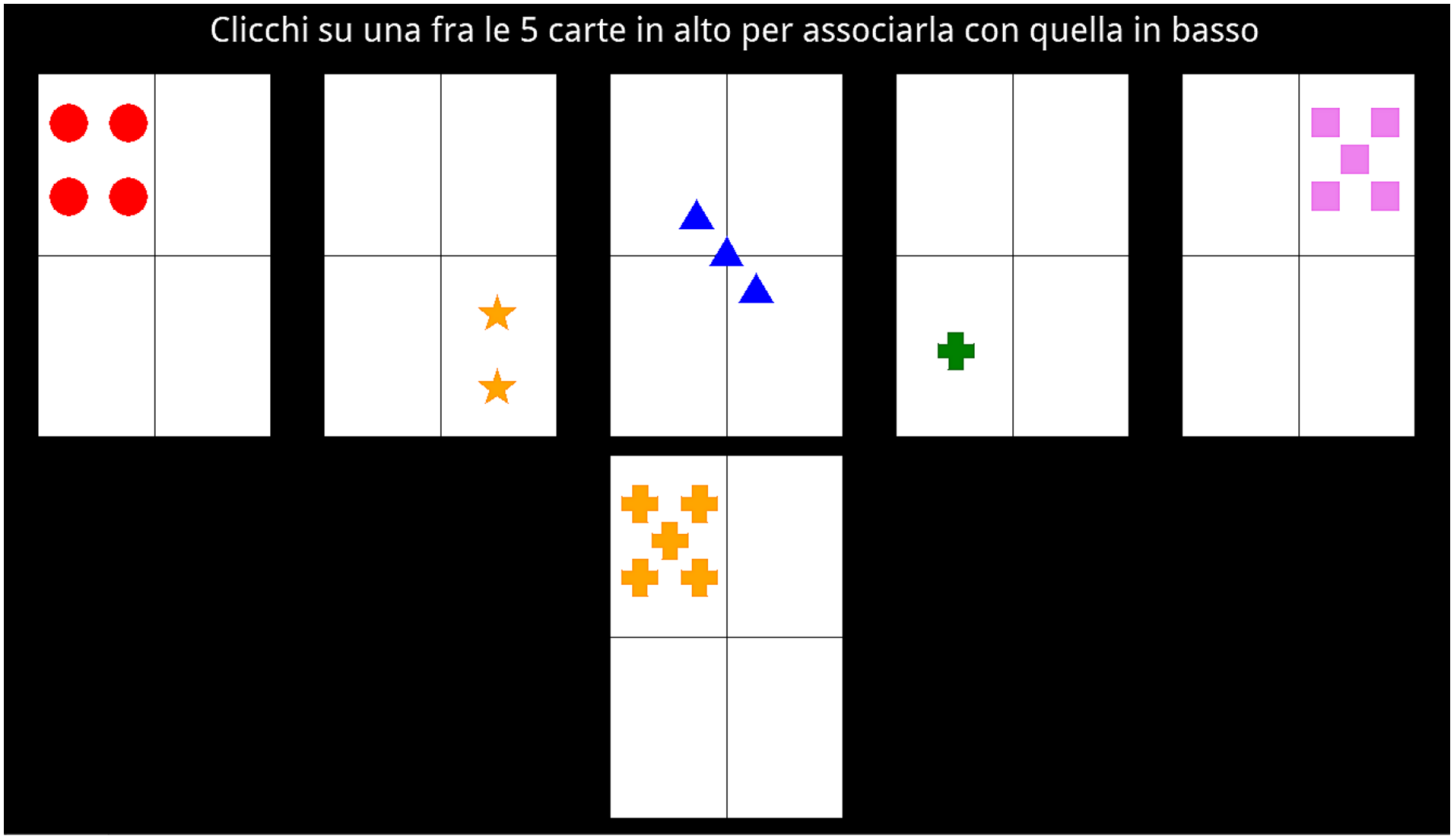
A screenshot from one example trial of the card sorting task. The text above says: “click on one of the 5 cards above to match it with the one below”.

To avoid the confounding effects of response-level learning (Steinke et al., 2020), on each trial, a different set of five choice cards was sorted and presented horizontally at the top of the screen in random order. There were no feature-level repetitions in each set of choice cards (e.g., only one card depicting the blue color). A reference card appeared simultaneously at the bottom of the screen. Participants were asked to match the reference card with one of the choice cards by clicking on the chosen one using the mouse and without temporal constraints. Participants had to guess which of four possible rules representing the four card dimensions (shape, color, number, or position) was currently relevant on each trial. The cards were arranged in such a way as to allow unambiguous evaluations of the used matching rule: each of the choice cards was matchable with each level of the four rules (shape, color, number, and position) plus a fifth card which was unmatchable with any of the four rules (Figure 1). If the participant’s choice was consistent with the relevant rule, the selected choice card became framed in green (i.e., positive feedback). Otherwise, it became framed in red (i.e., negative feedback). The feedback stayed on screen for 1 s. After each feedback, a new trial began.

Participants were not explicitly aware of the relevant rule on each trial; therefore, they were required to ground their choice on the last feedback received. Indeed, they were instructed to keep on matching cards with the same rule after positive feedback but change the rule after negative feedback. Furthermore, participants were informed that a rule might be relevant for a run of trials and unexpectedly change. The sequence of rules in the task was pseudo-random: the only constraint was that direct rule repetitions (from one run to the next) were not possible. Unknown to participants, the criterion for a change in the relevant rule was based on the number of consecutive positive feedback received. The criterion varied run-by-run to avoid participants anticipating the rule change. On each run, the criterion was randomly drawn from the inverse of an exponential distribution (scale = 2) with a minimum of 3 but no more than 6 correct trials (e.g., a minimum of 2 consecutive additional matches after the first trials leading to the identification of the relevant rule) to reach an acceptable trade-off between shifts and total possible trials. The task ended after participants completed 50 runs or performed 495 trials. Participants were explicitly instructed about the four rules and underwent a practice session to familiarize themselves with the task. In the practice session, the relevant rule was explicitly cued at trial onset, and the criterion was set to two consecutive correct matches for each of the four runs.

**Figure 2:**
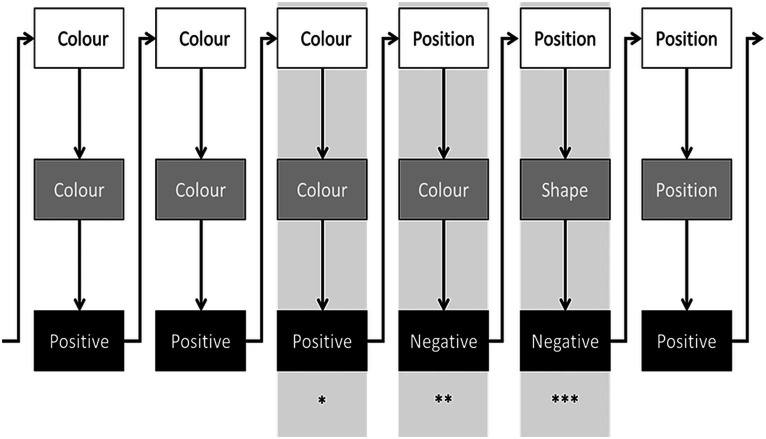
An example of a few experimental trials. White rectangles represent the relevant rule on each experimental run. Grey rectangles represent the rules used by a given participant. Black rectangles represent the feedback on each trial. Arrows depict the temporal succession of events. *Last trial of a run (colour in the example); **First negative feedback due to the change in the relevant rule (participants usually repeat the previous rule since the previous trial provided positive feedback); ***First trial after the negative feedback following a change in the relevant rule (i.e., the shift trial).

**Figure 3:**
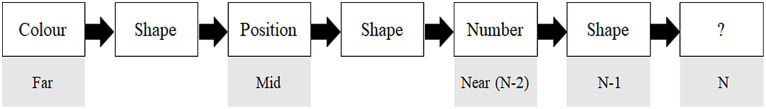
An example of a sequence of runs. White rectangles represent the relevant rule on each experimental run. After the rule change (last rectangle) participants do not know which is the relevant rule of the N run. At that point, they shift from the “Shape” run (N-1). The remaining three viable rules (“Number,” “Position,” and “Colour”) can be categorized according to the distance between the N run and the last run in which they have been relevant (“Near,” “Mid,” and “Far”). The nearest rule was always run N-2, while the other two may vary according to their sequential occurrence.

Since we were interested in the participants’ preference in rule selection, we considered the first trial after each negative feedback following a change in the relevant rule (hereafter, the shift trial; Figure 2). To test the hypothesis that such a preference is influenced by how recently the rules were relevant, we categorized rules selected by participants according to the distance between the current run (N) and the last run in which they were relevant. Specifically, on the shift trial, each of the three possible remaining rules was categorized as near (the most recent, always at the N-2 run), medium, and far (Figure 3). Therefore, while the N-3 lag could be only medium, more distant lags might be occasionally categorized into both medium and far levels according to the randomized sequence. The usage of the rule that participants should shift from (N-1) determines perseverations.

Finally, to control for possible unbalanced perceived occurrence among the four rules, we calculated the participants’ estimation of the rules’ occurrence separately for each of the four rules. At the end of the task, participants were prompted to estimate and report the number of times it was required to use each rule during the whole session.

### 2.3 Working memory span tasks

The shortened versions of three Working Memory Span tasks (Operation Span, Reading Span, and Symmetry Span; Oswald et al., 2015) were administered via a laptop to obtain a domain-general measure of the participants’ Working Memory Capacity (WMC). Each administration of the three tasks consisted of a processing component (a task to perform), a storage component (an element to be remembered), and a recall phase. Pairs of processing tasks (e.g., an arithmetic problem) and elements to be remembered (e.g., a letter) were presented sequentially. Participants completed a preliminary training first separately for the two components and then for the two components interleaved, with a fixed set size (e.g., number of problem-letter pairs) of 2. The mean time (+ 2.5 SD) spent by participants in responding to the processing task training determined a temporal constraint for each trial in the experimental phase.

#### 2.3.1 Operation span task

In the Operation Span Task (OSPAN), participants were presented with a set of arithmetic problems and were required to determine whether the provided solution was true or false. Following each problem, they were presented with a letter they had to retain and recall at the end of the set. Set sizes ranged between 4 and 6 with two administrations for each one.

#### 2.3.2 Reading span task

In the Reading Span Task (RSPAN), participants were presented with a set of sentences whose veracity they had to judge. Such as the OSPAN, sentences were interleaved with letters they had to retain for recall at the end of each set. Even in this case, set sizes ranged between 4 and 6 with two administrations for each one.

#### 2.3.3 Symmetry span task

In the Symmetry Span Task (SSPAN), participants were shown 8×8 matrices of black and white squares and had to judge whether each matrix of the set was symmetrical or not. In this task, the element to be retained for recall at the end of each set size was the position of a red square in a 4 × 4 matrix. Set sizes ranged between 3 and 5 with two administrations for each set size.

### 2.4 Procedure

After signing the informed consent, participants were instructed and administered the WM span tasks and the Card Sorting Task. About half of the participants started with the Card Sorting Task and the other half with the WM span tasks. All the tasks were administered in a quiet environment without distractions. Due to COVID-related restrictions, part of the sample (N = 11) was tested by the experimenters at participants’ homes, while the remaining part was tested in laboratory rooms with the same setup and device.

### 2.5 Statistical analyses

The frequency of rules’ use after a shift was calculated as the proportion of “perseverations,” “near,” “mid,” and “far” choices. Intending to inspect effective rule-shifting behavior, we also measured decision times as the time spent by the subject in selecting a choice card (from the trial onset to the card selection), separately for participants’ correct rule repetitions (using the same rule after a positive feedback) and rule efficient shifts (changing the rule after a negative feedback). In addition, we calculated error measures (Lange et al., 2016). Perseveration errors (PE) were calculated as the percentage of participants’ rule repetition in shift trials, upon the total number of responses in shift trials, excluding anticipatory shifts (i.e., when participants guess the shift of the rule in advance, before the negative feedback). Set loss errors (SE) were calculated as the percentage of participants’ rule shifts after positive feedback following at least two correct sorts (divided by the total number of responses after two consecutive correct sorts, i.e., repetition trials). Integration errors (IE) were calculated as the percentage of errors in integration trials. Integration trials may occur if the participant after a rule change has tried all but one viable rule in succession. In our task, this situation would occur on the third trial following the rule change. Therefore, in an integration trial, there would be only one rule left to try, which by definition would be the correct one. In integration trials, integration errors occurred when participants tried to use again a rule that had already resulted in negative feedback.

A WM span score for each task was calculated using the item-level partial credit unit method (Conway et al., 2005) and transformed into z-scores. Then, the three *z*-scores were averaged to obtain a WM composite score. The adoption of a composite score was intended to control for individual differences in WM capacity across heterogeneous contents. Participants were prompted to maintain a high average level of accuracy (>85%) in the processing task during the performance. We decided to exclude the WM scores for participants who performed less than 70% in the processing task. Average proportions of used rules as a function of their relevance recency (perseverations, near, medium, far) were analyzed through a one-way repeated-measures ANCOVA, with the WM composite score included as a continuous covariate. Additionally, error percentages were analyzed in a one-way repeated measures ANCOVA with Error type (PE, SE, and IE) as a within-subjects factor. The WM composite score was included as a continuous covariate to control through a direct behavioral measure the impact of interindividual differences in WMC on the different error types (Lange et al., 2016). Pearson correlations between the WM composite score and each of the error measures were also conducted. The participants’ estimated relative occurrence of the four rules during the task was analyzed in a one-way ANOVA with Rule (shape, color, number, position) as a repeated-measures factor. The arcsine-square-root transformation (Sokal and Rohlf, 1995) was applied to all the average proportions before the analyses. Finally, as a performance check, decision times to correctly repeat a rule and to correctly shift the rule were compared using a paired *t*-test, testing if rule shifts were slower than rule repetitions.

## 3 Results

As regards the WMC measures, mean accuracies in the processing tasks were 92.16, 92.21, and 93.93% for the OSPAN, RSPAN, and SSPAN tasks, respectively. One participant did not reach the 70% accuracy threshold for the SSPAN task and for that participant, the WMC score was calculated as the mean between the remaining two. Paired *t*-tests on the WMC z-scores did not show any statistically significant difference among the three tasks, *t*(28) = 0.00, *p* = 1; *t*(27) = 0.24, *p* = 0.81; *t*(27) = 0.02, *p* = 0.99 for the comparisons between OSPAN and RSPAN, between OSPAN and SSPAN, and between RSPAN and SSPAN, respectively.

**Figure 4:**
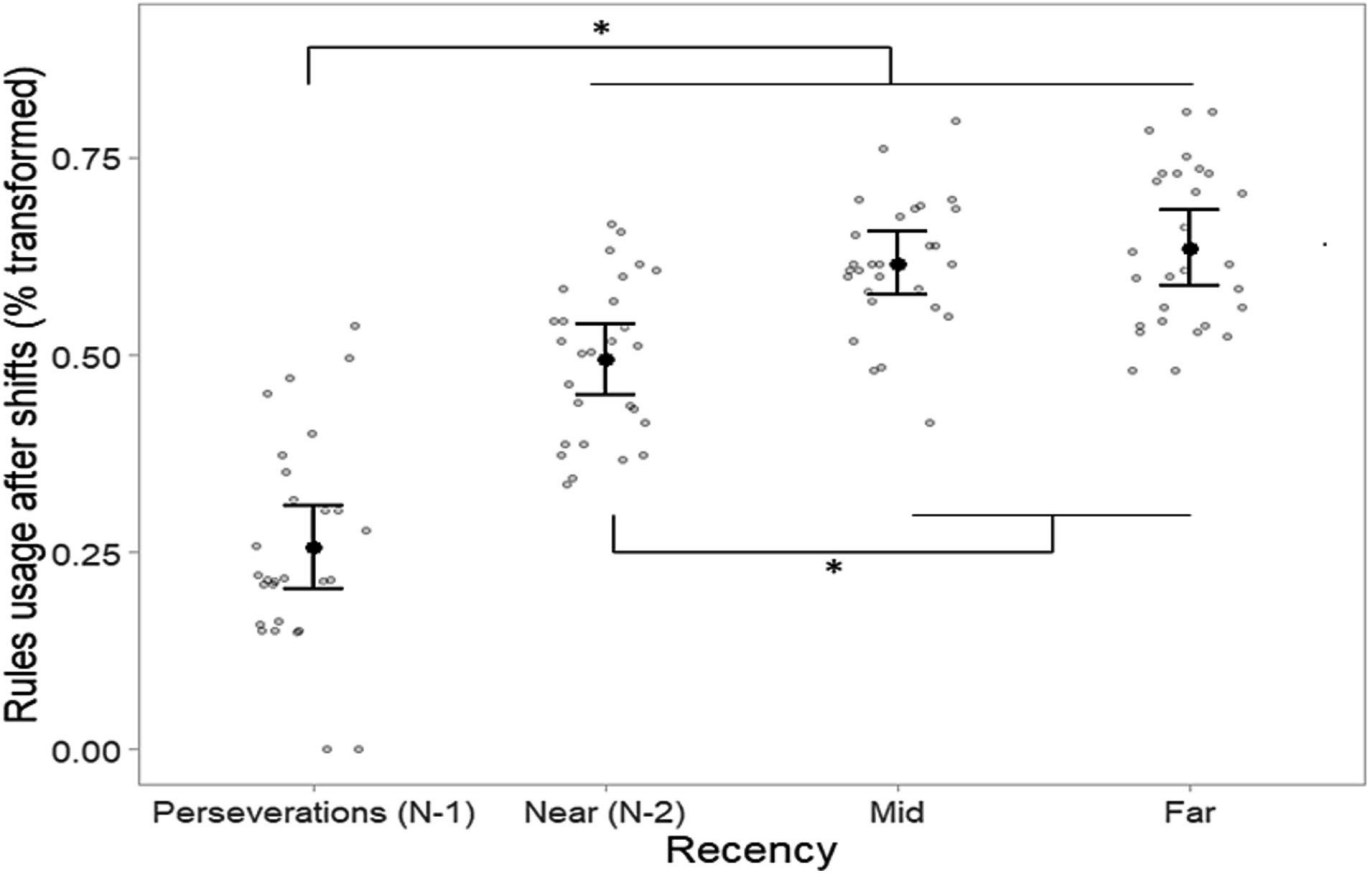
Proportions of used rules (arcsine-square-root transformed) as a function of their relevance recency in the first trial after a change in the relevant rule. Rules categorized as “Near” were always relevant in the N-2 run. Error bars denote the 95% Cousineau-Morey confidence interval for repeated measures (Cousineau, 2005; Morey, 2008) as implemented in the R package *superb* (Cousineau et al., 2021). * = *p* < 0.01.

One participant did not achieve the 50 categories in the card sorting task within the maximum number of trials allowed and was therefore excluded from the subsequent analyses. The remaining participants took an average of about 18 min to complete the sorting task with an average of 334.7 sorts (SD 40.9). For the analysis of the rules usage after shifts, we excluded trials in which participants anticipated the shift (shifting the rule at the right time but without the occurrence of negative feedback) as well as the shift trials occurring before each of the four rules became relevant at least one time. The average number of analyzable trials was 42.7. The WM span covariate exhibited no statistically significant interaction with the Recency factor, *F*(3, 78) = 1.05, *p* = 0.37, η^2^_p_ = 0.04, nor showed a significant main effect, *F*(1, 26) = 1.36, *p* = 0.25, η^2^_p_ = 0.05, in the main ANCOVA and was therefore removed. The Mauchly sphericity test revealed no significant violation of sphericity, *W* = 0.86, *p* = 0.56, and the ANOVA revealed the significant effect of the Recency factor, *F*(3, 81) = 57.96, *p* < 0.001, η^2^_p_ = 0.68. Tukey *post-hoc* tests showed that the rules that were relevant at run N-2 (i.e., Near condition) were less used after a rule shift than rules that were less recently relevant, *t*(27) = −4.51, *p* < 0.001, *d* = −0.85, for the contrast between Near and Mid, and *t*(27) = −4.17, *p* = 0.002, *d* = −0.79 for the one between Near and Far. Conversely, the difference between the Mid and Far rules was not statistically significant, *t*(27) = −0.7, *p* = 0.90, *d* = −0.13. Therefore, participants showed a reduced preference for recently abandoned rules than less recently abandoned ones (Figure 4). As expected, perseverations (N-1) were significantly lower (*p* < 0.001) than all the other recency conditions, *t*(27) = −7.02, *d* = −1.33; *t*(27) = −10.49, *d* = −1.98; *t*(27) = −10.59, *d* = −2.00 for the comparison with Near, Mid, and Far, respectively. Untransformed averages were 7.87, 23.07, 33.55, and 35.51% for the use of N-1, Near, Mid, and Far rules, respectively.

The mean integration trials computed for the calculation of IEs was 13.75 (SD 3.75), the mean repetition trials computed for the calculation of SEs was 145.29 (SD 9.76), and the mean shift trials computed for the calculation of PEs was 47.43 (SD 2.7). Participants committed an average of 0.46 (SD 0.74, max = 3) total “None” errors (i.e., sorted the unmatchable card). In the analysis of transformed proportions of IEs, SEs, and PEs, sphericity was significantly violated, *W* = 0.38 *p* < 0.001, and Greenhouse–Geisser correction was applied to the results. The WM composite score did not significantly interact with the Error type factor, *F*(1.231, 32.012) = 1.74, *p* = 0.2, η^2^_p_ = 0.06, but, since it produced a significant main effect, *F*(1, 26) = 5.77, *p* = 0.02, η^2^_p_ = 0.18, we decided to keep it into the main analysis. The analysis showed a significant main effect of Error type, *F*(1.231, 32.012) = 51.21, *p* < 0.001, η^2^_p_ = 0.66, revealing that IEs were significantly more frequent than both PEs, *t*(26) = 5.89, *p* < 0.001, *d* = 1.65 and SE, *t*(26) = 8.74, *p* < 0.001, *d* = 1.11. PEs were also significantly more frequent than SEs, *t*(26) = 5.9, *p* < 0.001, *d* = 1.11. Therefore, participants committed more errors in integration trials than the other two types of errors (Table 1), replicating previous results (Lange et al., 2016). Pearson’s correlations showed that the WM composite score was moderately and significantly correlated to SEs, but not significantly correlated to IEs and PEs (Table 2). Taken together, correlations showed that a measure of WM span is mostly associated with rule maintenance in card sorting tasks (Lange et al., 2016).

**Table 1:** Untransformed means and descriptive statistics for the proportion of errors.

Error type	Mean	SE	Min	Max
% PE	7.66	1.25	0	26.09
% SE	3.08	0.62	0	14.54
% IE	29.12	3.83	0	80.00

**Table 2:** Correlation matrix among the transformed proportion of errors and WMC composite *z*-score.

		WMC score	PE	SE	IE
WMC score	Pearson’s r	—			
	*p*-value	—			
PE	Pearson’s r	−0.243	—		
	*p*-value	0.213	—		
SE	Pearson’s r	−0.481	0.646	—	
	*p*-value	0.01	< 0.001	—	
IE	Pearson’s r	−0.364	0.377	0.56	—
	*p*-value	0.057	0.048	0.002	—

For the analysis of decision times, those faster than 300 msec, as well as those slower than 3 standard deviations above the sample mean, separately for repetitions and shifts, were considered outliers and removed. Participants’ rule repetitions after positive feedback (*M* 1,685 ms, *SD* 310 ms) were significantly faster than rule shifts after negative feedback (*M* 2286 ms, *SD* 556 ms), as shown in the analysis of decision times, *t*(27) = 7.46, *p* < 0.001, *d* = 1.41. Therefore, participants exhibited a reliable increase in ponderation time when shifting the chosen rule after negative feedback. Finally, no indication of an unbalanced perceived occurrence of the four rules was observed (means were 26.07, 24.86, 24.27, and 24.80% for the occurrence estimation of the color, numerosity, position, and shape rules, respectively). The analysis of the (transformed) subjective estimation of the four rules occurrence provided at the end of the task exhibited a significant violation of the sphericity assumption, *W* = 0.47, *p* = 0.002, and the Greenhouse–Geisser correction was applied to the results. No unbalanced estimation of the occurrence of the rules was found since the effect of Rule was not statistically significant, *F*(2.032, 54.866) < 1, *p* = 0.72, η^2^_p_ = 0.01.

## 4 Discussion

In the present study, we tested the hypothesis that inhibition, known to affect task switching performance under cued task set switching, also affects rule selection (i.e., the probability of a specific task set rule to be selected) under uncertainty. If decision-making processes underlying rule inference and selection are influenced by the sequential history of rule application, then the levels of activation of currently irrelevant rules are not equivalent but may vary depending on their recency of use. In forced-choice task-switching, task-set inhibition determines slower RTs and increased ERs when participants must perform recently abandoned tasks (ABA) than when they are required to perform less recently abandoned tasks (CBA). We searched for this N-2 repetition cost in free-choice behavior in an implicit rule-shifting paradigm by considering how many runs of trials since the last run in which a given rule was relevant. We found that participants were less likely to try the sorting rule that was relevant two runs before the current run (N-2) compared to the ones that were relevant further runs before. Assuming a persisting inhibition account (Koch et al., 2010), these results indicate that inhibitory control, known to affect the speed and accuracy of task set implementation, may also modulate the probability of a specific task set rule being selected for guiding future actions. In line with previous results (Schuch and Dignath, 2021), our results may suggest that proactive interference may cue decision-making not to select actions which have been subject to such interference. With our work, we expanded the current knowledge about the management of cognitive interference in task-switching and rule-shifting providing evidence that conflict-related mechanisms may also affect the processes involved in rule inference and selection.

Research studying the link between cognitive control and decision-making indeed suggested that two different behavioral strategies may be triggered by conflict situations: namely, conflict adjustment and conflict avoidance (Dignath et al., 2015) described in an integrative framework of conflict adaptation (Botvinick, 2007). Conflict adjustment refers to top-down compensation having the functional role of protecting task performance after a conflict by transiently strengthening task-relevant features (e.g., the Gratton effect, Gratton et al., 1992). Differently, conflict avoidance refers to an anticipatory disengagement from more demanding activities and tasks (e.g., Arrington and Logan, 2004; Kool et al., 2010), which is suggested to be proactively controlled and operates on a longer timescale, involving learning processes (Botvinick, 2007; Braver et al., 2009; Dignath et al., 2015). However, in our paradigm, the conflicting dimension is not particularly associated with one specific rule (e.g., with the shape rule) but occurs on a trial-by-trial basis (Dignath et al., 2015). Therefore, the flexible mechanisms of rule inference and selection seem to reflect the existence of an anticipatory control strategy capable of dealing with the competing rules maintained in a pending state, as the cognitive branching account suggests (Koechlin and Hyafil, 2007; Charron and Koechlin, 2010). Indeed, once the rule selected for execution provides negative feedback, one of the alternative pre-active rules must be chosen. If a negative recency bias in the selection exists, it may be due to the inhibition or reduced pre-activation of the most recently relevant rule. Such an inhibitory mechanism may in turn trigger avoidance in rule inference and selection. We showed that this conflict avoidance may persist over a series of events (i.e., runs of trials), consistently with the view that inhibition decays over time (Meiran, 2000).

A possible alternative explanation of our results would imply the involvement of probabilistic choice behavior during rule inference. Indeed, the pattern observed when participants guessed the next relevant rule seems to reflect the alternation bias which would be predicted by the representativeness heuristic (Tversky and Kahneman, 1974). However, this contrasts with the finding commonly observed in voluntary task-switching paradigms, where a repetition bias seems to prevail instead (Arrington and Logan, 2005). It has been suggested that in voluntary task-switching paradigms, a competition between representativeness and availability may occur for choice probability (Arrington and Logan, 2005; Vandierendonck et al., 2012). However, it’s important to note that similar patterns have been mostly observed in paradigms consisting of two tasks. In a recent study, Schuch and Dignath (2021) showed that task selection among three tasks can be biased away from conflicting tasks (in ABA sequences) and have excluded an interpretation based on a randomness heuristic. Indeed, when participants were not required to perform the task but only to decide the next cue (color) in the sequence, the task selection bias was not observed (Schuch and Dignath, 2021). In addition, other evidence has shown that the backward inhibition and the N-2 repetition also occur in unspeeded tasks (Foti et al., 2015), suggesting the ubiquity of this mechanism.

### 4.1 Feedback-driven conflict and the role of WM

Since performance in WCST-like tasks is guided by the experience of feedback, it is conceivable that the conflict between the task rule that the subjects were using and the changed state of the environment is triggered by the occurrence of negative feedback. Arguably, the rise of the feedback-driven conflict would instantiate the inhibition of the abandoned rule representation, in order to not perseverate (keep on sorting with the same incorrect rule). In this regard, the tendency to not reuse such representation would suggest the existence of persisting inhibition. Interestingly, Zanolie et al. (2008) have found that the informativeness of feedback distinctively activates two brain areas involved in feedback processing, the dorsolateral prefrontal cortex (DLPFC) and the anterior cingulate cortex (ACC). The DLPFC has a prominent role in goal-directed behavior (Miller and Cohen, 2001) and in the active maintenance of task sets (Yeung et al., 2006); the ACC is pivotal in models of behavioral conflict-related adaptation, given its largely known role in conflict monitoring and outcome evaluation (Botvinick, 2007). Relatedly, the authors (Zanolie et al., 2008) observed greater activations in the ACC following the first negative feedback acting as a rule change signal than following subsequent efficient errors (e.g., with the second negative feedback), while the DLPFC exhibited higher activation following efficient errors compared to the ACC (Zanolie et al., 2008).

In WCST-like tasks, when required to shift the sorting rule, participants must infer the next correct rule. While the probability of being correct on the first try is random, such a probability would decrease after the first efficient error depending on the number of viable rules. Accordingly, errors in rule inference at this stage (i.e., attempting to match a card according to a sorting rule which has been already shown to be wrong), have been shown to increase in older individuals and with the increase in the number of viable rules (Lange et al., 2016) suggesting, even if indirect, a link between WM load and capacity with rule inference abilities. In the present work, we obtained a behavioral measure of WMC and found that it is negatively correlated to set maintenance (SEs) and negatively but marginally correlated to rule inference abilities (as measured by IEs). Coherently with Lange et al. (2016), we found that WMC was not correlated to set-shifting abilities, as measured by PEs. Conversely, we found that individuals’ WMC does not apparently play a role in the biased tendency to not attempt to match with recently relevant rules under high uncertainty. Such an apparent lack of relationship may suggest that, while the capacity limits of the WM may play a role in the abilities to actively maintain and infer rules (Lange et al., 2016), activation levels of pre-active rules determining biases in rule selection can be unaffected. Nevertheless, the association between WMC and rule inference and selection processes requires further investigation, involving, for instance, other populations or experimental conditions.

## 5 Conclusion

In conclusion, the findings of the present study jointly suggest that (a) rule inference under uncertainty (after a rule shifting) is biased toward the systematic avoidance of reusing rules that have been recently relevant and (b) working memory capacity, despite its relationship with inefficient non-perseverative errors, seems not to play a role in such a tendency.

Extending the implications of the use of card-sorting tasks for the assessment of executive processes and cognitive flexibility as has been already described (Lange et al., 2016), our results suggest that these tasks can be valuable in evaluating inhibitory mechanisms in choice behavior. While further research is needed to inspect the generalization of these results to other populations (both healthy and pathological) and experimental conditions, the results it provides are promising. Indeed, clinical testing can be improved by the assessment of decision-making and rule inference under uncertainty since this area may reflect changes that macroscopic measures, such as PEs, cannot detect. Indeed, while the observed bias may be the outcome of a functioning inhibitory process, its absence may set off an alarm bell and start the project of targeted cognitive and neuropsychological training.

## Data availability statement

The raw data supporting the conclusions of this article will be made available by the authors, without undue reservation.

## Ethics statement

The studies involving humans were approved by Institutional Review Board of the Department of Psychology, Sapienza University of Rome. The studies were conducted in accordance with the local legislation and institutional requirements. The participants provided their written informed consent to participate in this study.

## Author contributions

PZ: Conceptualization, Data curation, Formal analysis, Methodology, Software, Writing – original draft. AZ: Conceptualization, Investigation, Writing – original draft. AC: Conceptualization, Supervision, Writing – review & editing. FF: Conceptualization, Supervision, Writing – review & editing. SS: Conceptualization, Supervision, Writing – review & editing.
